# Host immunity, nutrition and coinfection alter longitudinal infection patterns of schistosomes in a free ranging African buffalo population

**DOI:** 10.1371/journal.pntd.0006122

**Published:** 2017-12-18

**Authors:** Brianna R. Beechler, Anna E. Jolles, Sarah A. Budischak, Paul L. A. M. Corstjens, Vanessa O. Ezenwa, Mireya Smith, Robert S. Spaan, Govert J. van Dam, Michelle L. Steinauer

**Affiliations:** 1 College of Veterinary Medicine, Oregon State University, Corvallis, OR, United States of America; 2 Department of Integrative Biology, Oregon State University, Corvallis, OR, United States of America; 3 Odum School of Ecology, University of Georgia, Athens, GA, United States of America; 4 Department of Molecular Cell Biology, Leiden University Medical Center, Leiden, The Netherlands; 5 College of Veterinary Medicine, University of Georgia, Athens, GA, United States of America; 6 Department of Fisheries and Wildlife, Oregon State University, Corvallis, OR, United States of America; 7 Department of Parasitology, Leiden University Medical Center, Leiden, The Netherlands; 8 College of Osteopathic Medicine of the PNW, Western University of Health Sciences, Lebanon, OR, United States of America; Case Western Reserve University School of Medicine, UNITED STATES

## Abstract

Schistosomes are trematode parasites of global importance, causing infections in millions of people, livestock, and wildlife. Most studies on schistosomiasis, involve human subjects; as such, there is a paucity of longitudinal studies investigating parasite dynamics in the absence of intervention. As a consequence, despite decades of research on schistosomiasis, our understanding of its ecology in natural host populations is centered around how environmental exposure and acquired immunity influence acquisition of parasites, while very little is known about the influence of host physiology, coinfection and clearance in the absence of drug treatment. We used a 4-year study in free-ranging African buffalo to investigate natural schistosome dynamics. We asked (i) what are the spatial and temporal patterns of schistosome infections; (ii) how do parasite burdens vary over time within individual hosts; and (iii) what host factors (immunological, physiological, co-infection) and environmental factors (season, location) explain patterns of schistosome acquisition and loss in buffalo? Schistosome infections were common among buffalo. Microgeographic structure explained some variation in parasite burdens among hosts, indicating transmission hotspots. Overall, parasite burdens ratcheted up over time; however, gains in schistosome abundance in the dry season were partially offset by losses in the wet season, with some hosts demonstrating complete clearance of infection. Variation among buffalo in schistosome loss was associated with immunologic and nutritional factors, as well as co-infection by the gastrointestinal helminth *Cooperia fuelleborni*. Our results demonstrate that schistosome infections are surprisingly dynamic in a free-living mammalian host population, and point to a role for host factors in driving variation in parasite clearance, but not parasite acquisition which is driven by seasonal changes and spatial habitat utilization. Our study illustrates the power of longitudinal studies for discovering mechanisms underlying parasite dynamics in individual animals and populations.

## Author summary

Schistosomes are a parasite of global importance, affecting over 200 million people worldwide, while also infecting livestock and wildlife. Despite decades of research on schistosomiasis in humans, little is known about what drives patterns of infection in untreated naturally occurring populations. We took advantage of a study in African buffalo to understand how geography, nutrition, immunity and coinfection drive schistosome acquisition and loss. The most striking outcome of our study was that schistosome burden varied seasonally within an individual, with some hosts able to completely clear infection, and others unable to do so. The ability of a buffalo to clear infection was affected by immune response and co-infection with other gastrointestinal parasites while host immunity and coinfection were not important in determining whether a buffalo became infected. These outcomes should be taken into consideration when designing control programs for human schistosomiasis.

## Introduction

Schistosomes are a diverse, globally important group of trematode parasites that cause chronic inflammatory disease in humans, livestock and wildlife. They infect over 200 million people worldwide[1], and inflict significant morbidity on already struggling human populations[2–4]. Several species of schistosomes infect livestock such as cattle, sheep and goats and cause significant economic loss and loss of valuable protein in regions where malnutrition is widespread[5–9]. Schistosomes are also parasites of wildlife including charismatic megafauna such as elephant, hippopotamus, rhinoceros, and African buffalo[10–13]. Although the effects of these parasites on wild animal populations are largely unknown, infection can cause severe pathology, and is a concern for conservation efforts[14,15].

Like most parasites, schistosome infection levels are highly heterogenic among individuals[16,17]. Based on decades of studies on human populations, variation in worm burdens is mainly attributed to the interplay of two factors: exposure and immunity[18]. Because schistosomes are transmitted to hosts through contact with aquatic habitats where their snail vectors thrive, exposure is determined by host behavior that brings the hosts into contact with snail-infested water sources and environmental factors that increase snail density (e.g.[19]). Indeed, infection is typically clustered spatially into hotspots that are associated with water bodies, especially those that provide excellent habitat for snail vectors (e.g.[1,17,20–22]). Decades of continued exposure to schistosomes slowly drives acquired immunity to reinfection; however, resistance is typically not complete[23–27] and is associated with increased eosinophils (a white blood cell important in immune response to multicellular parasites)[28] and specific IgE antibodies (antibodies important in response to multicellular parasites)[23–27]. Even with the same exposure levels, the development of immunity is heterogenic among individuals[29], which could be driven by underlying genetic differences in immune genes[30] or other factors that affect immune function such as host condition or co-infection.

Despite the rich literature linking environmental exposure and immunity with schistosome infection, other ecologic factors largely have been unexplored. Theoretical and empirical studies suggest that resource availability and the presence of co-infecting pathogens are likely to influence schistosome dynamics[31,32]. Resource availability (i.e. food intake) influences both the host immune response and pathogen fitness. Thus, increasing resources are predicted to have both costs and benefits for the pathogen[33–35]. For example, if host immunity is energetically costly, hosts in poor condition (low resource acquisition) would have a limited immune response and thus be more susceptible to infection while those in good condition (high resource acquisition) would have higher immune clearance[31,33,34]. Thus, in this scenario, low resource acquisition of the host would benefit the parasite. On the other hand, if parasite success is dependent on host resource acquisition, hosts in good condition will provide more resources for parasite growth and thus their parasites will be more successful[34,36]. This success could increase longevity of infection and also increase infective stages released into the environment; therefore, increasing within-host burdens over time. Thus, there is a dynamic interplay between resource availability, host immunity, and pathogen success. Indeed, this type of dynamic has been demonstrated in other host-pathogen systems, such as in gut nematodes of the Cuban tree-frog where resource restriction altered the host response to the pathogen, with the host employing a "tolerance" strategy when allowed to eat freely and using a "resistance" strategy when food-restricted [37]. Resource availability is therefore likely to have strong impact on schistosome dynamics because acquired immunity is a strong regulator of worm burdens and schistosomes are affected by host diet deficiency[38–42].

Multiple species of parasites within a host are also likely to influence the recruitment and disease course of new parasites, which in turn can impact disease dynamic patterns[43–45]. These parasites could compete directly within the host or through the immune system of the host, limiting or enhancing each other’s recruitment and maintenance[46–48]. Schistosomes are well-known to influence the disease course of other pathogens in experimental models, changing both host susceptibility and pathology due to other pathogens via effects on the host immune system[32,49,50]. However, less is known about how other pathogens affect schistosome burdens, even though schistosomes commonly occur with many other helminth species (e.g.[51]). As part of their strategy to maintain a chronic infection, many helminths have multiple mechanisms to modulate the immunity of their hosts including general suppression through inhibition of Toll-like receptors (innate immune sensing) and the induction of regulatory T cells[52,53]. Furthermore, helminths may polarize the immune response in one direction, thereby preventing alternative responses (e.g. Th1/Th2 polarization). This has been demonstrated in African buffalo where helminths alter the progression of bovine tuberculosis by skewing the host immune response to a Th2 dominated response, rather than a Th1 response that is more effective at suppressing tuberculosis infection[44].

Another gap in the natural history of schistosome infection is determining the factors that influence the natural *loss* of parasites[54]. This parameter is not typically considered in human populations because chemotherapy usually follows diagnosis. Additionally, the importance of worm loss is perhaps overlooked because of the estimated longevity of infections (3–9 years) [55–57]. However, seasonal incidence of schistosomes in more temperate regions suggests a significant amount of worm loss on a shorter time scale[58,59]. Although largely over-looked, worm loss is an important parameter for host heath and disease dynamics. Worm burden directly influences disease pathology and the rate of worm loss is thought to enhance acquired immunity[29]. Thus, knowing the factors that drive the loss of established worms from a host has important implications.

In this paper, we take a novel approach, focusing on dynamics of schistosome infection within individual hosts over time, by separating ACQUISITION and LOSS of schistosomes in a longitudinal study design. We dissect effects of immunology, co-infections, environmental factors (spatial and seasonal), and host condition (as a measure of resource acquisition) on schistosome gain and loss in a free ranging African buffalo population. In southern Africa, African buffalo undergo extreme variation in resource availability due to seasonal variation in rainfall and temperature[60,61]; are known to be infected with numerous parasites and pathogens (e.g.[62–66]); and are long-lived, making longitudinal studies of parasite dynamics possible[67,68]. We ask: (i) What are the spatial and temporal patterns of schistosome infections in our study population; (ii) how do parasite burdens vary over time within individual hosts; and (iii) what host, co-infection, and environmental factors explain patterns of schistosome gain and loss in the buffalo? We find that schistosome populations are seasonally dynamic and acquisition of worms is primarily driven by exposure, while loss of worms is complex and is driven by immunology, host physiology and coinfections.

## Materials and methods

### Ethics statement

All animal procedures were approved by Oregon State University (ACUP 4478, ACUP 3267) and the University of Georgia (A2010 10-190-Y3-A5) Institutional Animal Care and Use Committees (IACUC), which follow the 8th Edition of the Guide for the Care and Use of Laboratory Animals (Guide), NRC 2011; the Guide for the Care and Use of Agricultural Animals in Research and Teaching (Ag Guide), FASS 2010; and the European Convention for the Protection of Vertebrate Animals Used for Experimental and Other Scientific Purposes, Council of Europe (ETS 123).

### Study site and capture

Kruger National Park (KNP) is located in northeastern South Africa and comprises approximately 19,000 km^2^, with an African buffalo (*Syncerus caffer*) population of approximately 30,000 animals[69]. We followed 200 buffalo in southern KNP between June 2008 and August 2012 to assess longitudinal variation in schistosome infection and determine the drivers of these changes–including the role of host nutrition, host immunity and coinfection with helminths (Table 1).

**Table 1:** Variables measured in African buffalo during the course of this study.

Environmental/ Demographic Variables	Nutritional Variables	Immune Variables	Disease Variables
Season of capture	Body Condition	Bactericidal ability of plasma	Nematode Fecal Egg Count
Year of capture	Total Plasma Protein	Total Serum Globulins	Schistosome burden (CAA concentration)
Location of capture	Fecal Nitrogen	Plasma cytokines (IFNy & IL4)	Bolus (anthelmintic received or not)
Micro-herd designation	Hematocrit	Total white blood cell count	Nematode species identification
Age		Eosinophil count	
Pregnancy Status		Haptoglobin	

Young adult female buffalo were captured from two herds and radio-collared in the southern portion of KNP, as part of a larger study on parasite interactions in free-ranging buffalo[44]. The first 100 buffalo were captured between June 23 and July 5 2008 (Lower Sabie herd) and the second 100 buffalo were captured between October 1 and October 8 2008 (Crocodile Bridge herd). During the study, any animal that died or migrated out of the study area was replaced by a similarly aged individual (n = 112), resulting in a total of 312 individual buffalo that were followed for a period ranging from six months to four years (median follow time was 3 years) with 1750 data points.

Animals were chemically immobilized with M99 (etorphine hydrochloride) and ketamine by darting from a helicopter or vehicle every 6 months for a maximum of 9 captures per individual. Following data collection, immobilization was reversed using M5050 (diprenorphine) and animals returned to their free-living existence. All immobilizations were performed by South African National Parks (SANParks) veterinarians and game capture staff.

### Environmental and demographic parameter assessment

After immobilization, demographic data were collected including: season of capture, age, pregnancy and lactation status and a long-acting anthelmintic drug (Panacur bolus -fenbendazole, Intervet UK) was applied to 50% of the individuals. This bolus reduced nematode burdens[44], but has no effect on schistosomes[70–72]. Season of capture was denoted as wet season (November to April) or dry season (May to October). Pregnancy was assessed by rectal palpation[73], which has a nearly 100% sensitivity rate after 51 days of gestation in Egyptian buffalo (Bos bubalis;[74]), while lactation was evaluated via manual milking of all 4 teats[75]. Age was assessed from incisor emergence patterns for buffalo 2–5 years old animals, and from tooth wear of incisor one for buffalo 6 years and older[76]. Location was determined at the time of initial capture. Both herds, Crocodile Bridge and Lower Sabie, utilized areas in the southern section of Kruger National Park. The Crocodile Bridge herd was comprised of seven (corner, mountain, malelane, mountain, powerline, randspruit, thicket) sub-herds which maintained separate home ranges but were connected by frequent inter-subherd dispersal and migration, however for the herd analyses in this study appropriate sample sizes only existed for 4 subherds (corner, mountain, powerline, randspruit)[77].

### Sample collection and parasite diagnostics

Blood was taken from each animal via jugular venipuncture between 15–45 minutes after darting. Blood was collected into no-additive tubes for harvesting serum, into heparinized tubes for harvesting plasma, and into EDTA additive tubes for whole blood. All samples were placed on ice in a cooler within 5 minutes of collection for transportation back to the laboratory. Serum and plasma were collected from the top of the appropriate blood tubes after centrifugation for 10 minutes at 5,000 g to ensure the separation of cytological components. Whole blood was mixed and aliquoted for storage. Total time between sample collection and sample testing or storage in appropriate conditions was never greater than 8 hours, and typically ranged between 4–6 hours. Blood samples not processed immediately were stored in microcentrifuge tubes at -20°C until analysis.

Schistosome infection was determined by a lateral flow assay to detect a specific schistosome antigen, circulating anodic antigen (CAA) in serum[78]. This antigen is produced by the schistosome and released into the blood[79]. The CAA concentration (pg/ml) was used as a proxy for adult worm burden in the buffalo, and has been shown to be highly sensitive and specific in human studies[80–84], as well as in cattle [85,86]. We validated it for use in buffalo by comparing counts of adult schistosomes at cull to CAA level (S1 Text). The species of schistosome was confirmed to be *Schistosoma mattheei* by sequencing a region of the large subunit ribosomal DNA and part of the mitochondrial DNA (S2 Text).

Feces was collected rectally from each animal, placed on ice and returned to the laboratory within 8 hours of collection. Gastrointestinal nematode infection was assessed using fecal egg counts. Fecal samples were processed on the day of collection using a modification of the McMaster method[87]. To identify the species of nematode worms present, feces were cultured to the infective larval stage and larvae were isolated using a modified Baerman apparatus, and then identified larve using PCR and sequencing[63]. The dominant helminths in this population of buffalo are gastrointestinal nematodes: *Cooperia fuelleborni*, *Haemonchus bedfordi* and *Haemonchus placei[63]*. The remaining feces was aliquoted and stored at -20°C or dried in a drying oven at 30°C for 24–48 hours. For a subset of the animals, captured at Lower Sabie in July and August 2009, part of the fresh sample (3 g) was processed via sedimentation to look for schistosome eggs[88]; however, due to the small amount of fecal material obtained via rectal collection, this was found to be an unreliable indicator of infection so was not continued.

### Measuring host nutrition and immunity

We expected host nutrition and immune response to play an important role in mediating schistosome dynamics within the host, so employed several parameters to assess each.

### Nutritional analyses

Body condition was measured by visually inspecting and palpating four areas on the animal where fat is stored in buffalo: ribs, spine, hips and base of tail. Each area was scored from 1 (very poor) to 5 (excellent) and a body condition score calculated as the average of all four areas. This index is correlated with the kidney fat index[89], and similar body condition indices have been used in other studies of African buffalo[90,91]. Fecal nitrogen, a measure of dietary protein[61,92] was assessed by the Agricultural Research Council (Nelspruit, South Africa) from dried feces[93]. Hematocrit, the volume percentage of red blood cells (the primary resource for adult schistosomes) in the blood, which is also correlated to body condition, was assessed by a hematology analyzer for whole blood on the day of capture[94]. Total plasma protein, a measure of animal nutritional status, was assessed using an Abaxis chemistry panel[95].

### Immune assays

We measured cytokine levels and cellular immune responses that are associated with Th1 and Th2 immunity from blood. Adaptive immunity against schistosomes in humans is associated with a Th2 response with increased levels of IgE antibodies and eosinophils. Therefore, we expected that resistance to schistosomes would be associated with high levels of IL4, total globulins, and eosinophils. We also expected markers associated with Th1 proinflammatory immunity (IFNy) to be low in these animals due to polarization toward Th2 responses. Finally, we evaluated nonspecific markers of innate immune function (plasma BKA) and inflammation (haptoglobin).

#### Immune assays—Cytokines (IL4 and IFNγ)

Cytokines are immunologically active proteins, that aid in cell signaling during a host immune response and have been proposed as an excellent way to simplistically and realistically describe the immune profiles[96]. We quantified IL4 and IFNy concentrations in whole blood in response to *in vitro* stimulation using pokeweed mitogen. Pokeweed is a general immune stimulant that is often used to induce cytokine and cell proliferation (e.g.[97]). Whole blood collected into lithium heparinized tubes was subdivided into 1.5 ml aliquots. Mitogen (15ug of pokeweed, Sigma L9379, rehydrated in 50 μl of PBS) was added and the mixture was incubated at 37°C for 24 hours. After 24 hours, plasma was removed and stored at -20°C until analysis. Levels of IFNy in the samples were assessed using a commercially available ELISA (Abd Serotec, product #MCA5638KZZ) following manufacturer instructions as described previously[44]. IL4 was assessed using a standard sandwich ELISA[98] with commercially available antibodies designed for bovines (Abd Serotec, CC308 and CC313) and recombinant IL4 for the standard curve (Abd Serotec, PBP010), as described in[99]. All samples were run in duplicate and the mean optical density was calculated for each set of duplicate wells at a wavelength of 405 nm. Sample concentrations for both cytokines were calculated using a linear standard curve and are expressed as pg/ml.

#### Immune assays—Plasma BKA

We assessed the ability of plasma to kill *Escheria coli* (modified from[73]). Plasma was diluted 1:3 with PBS, mixed with *E*. *coli*, and incubated with tryptic soy broth for 12 hours at 37°C. All samples were run on 96-well plates in duplicate as described by[100] and optimized for African buffalo. The percent of bacteria killed was calculated as: ((average absorbance in control well–average absorbance in sample well)/(average absorbance in control well)).

#### Immune assays—Total globulins

Total globulins (alpha, beta & gamma) were assessed using an Abaxis chemistry panel from samples stored at -20°C until the time of testing [95]. Time in storage did not affect the results (S3 Text).

#### Immune assays—Haptoglobin

Haptoglobin was evaluated from stored plasma samples (stored at -20°C) using a commercially available ELISA kit for bovine haptoglobin (Life Diagnostics, product number HAPT 11). Time in storage did not affect the results (S3 Text).

### Statistical analysis

#### Statistical analysis—Descriptive statistics

Animals were considered positive for schistosome infection if their CAA concentration was greater than 20 pg/ml, anything under 20 was considered negative (according to standard curves that were run simultaneously with the samples, S1 Text). Because of the highly aggregated infection pattern, CAA concentrations were natural log transformed which normalized the distribution, after the addition of a constant (1). Bolus status was accounted for in model selection, but was always eliminated as it explained little variance. Herd membership and 95% usage distribution were determined following previously described methods[101]. Briefly, satellite collars provided two locations daily (06:00 and 18:00 local time) to establish the general location of each buffalo herd. Once a herd was located, we visually verified presence/absence of collared individuals and recorded date, time and GPS location. For each herd, GPS locations for the period September 2009 to August 2012 were mapped in ArcMap 10.1 (Environmental Systems Research Institute, Inc., Redlands CA) and 95% utilization distributions estimated for each herd in the Geospatial Modelling Environment[101].

Model 1: To assess geographic patterns, seasonal patterns and the effect of year of the study a general linear mixed model was performed with Gaussian distribution and log link. The dependent variable was the log of CAA concentration, while the independent variables were binomial (season & herd-Lower Sabie vs Crocodile Bridge) or categorical (calendar year) with the random effect of Animal ID to account for repeat sampling of the same individual.

Model 2: Because significant geographic patterns were noted and due to the structure of the subherds at Crocodile Bridge[77] we did a further analysis evaluating whether subherd structure at Crocodile Bridge showed geographical pattern in schistosome burden using a similar GLMM but including only individuals at Crocodile Bridge, while including year and animal ID as random factors.

Model 3: To evaluate whether age correlated with schistosome burden we ran a GLMM with the same structure as model 1 but replacing year with age (to reduce covariance between year and age). Since age was a continuous variable we transformed it, dividing by 2 standard deviations[102], which places the mean at 0.5 and allows the numerical variables to be on the same scale as the binomial variables of season and herd of origin (LS vs CB).

#### Statistical analysis—Variables associated with a change in schistosome infection over time

The change in schistosome burden (change in CAA concentration) was calculated as the difference between burdens at consecutive collection time points, but only included animals that tested positive at least once in their lifetime (CAA>20). Also, animals with less than 4 time points of data were excluded, which resulted in 734 data points. Thus, only net gain and loss of worms were assessed, and it is possible that some degree of loss is masked by gain and vice versa. Change in schistosome burden was calculated over consecutive collections (approximately 6 months) and was associated with driver variables that were measured at the earlier time point. Thus, for example, those variables measured at T_0_, were associated with changes in burdens between T_0_ and T_1_. We ran 3 different models, the first (model 4) was to understand why some animals gained and some lost schistosomes, with the dependent variable being binomial (loss vs gain). We then ran two models, one for animals that had a net increase in schistosomes (model 5) and one that had a net decrease in schistosomes (model 6) to understand what variables predicted the degree to which animals lost or gained worms. Model 7 was run to clarify the role of gastrointestinal nematodes in schistosome loss.

Model 4: Difference in schistosome burden between consecutive time points (change in concentration) was classified as gain (increase) or loss (decrease). We then ran a generalized linear mixed model (binomial distribution, logit link) with random terms of animal ID, to account for repeated sampling, and subherd, to account for geographic variation, to look at factors associated with net gain and loss.

Models 5 & 6: To further evaluate the magnitude of schistosome gain and loss we created two separate linear models, one for net increase (model 5) and one for net decrease (model 6). To calculate the change, we simply subtracted the animals previous burden (at the prior 6-month time period, T-_1_) from its current burden. Therefore, the dependent variable is numeric (change in concentration, and was then log transformed for normality. We again used random terms of animal ID and subherd.

Model 7: In buffalo the community of gastrointestinal nematodes is dominated by *C*. *fuelleborni*, *H*. *bedfordi* and *H*. *placei[63]*. We ran model 7 similar to model 6, except the variable nematode was replaced with two variables, presence/absence of *Haemonchus* and presence/absence of *C*. *fuelleborni*. Including this variable decreased our sample size to n = 195, so this model was performed separately–simply replacing nematode fecal egg count with these 2 variables.

#### Statistical analysis—Description of full models and model selection

For models 4–8 we considered 8 predictor variables at the time previous time point (T-_1_) and all two way-interaction terms. All continuous variables were transformed to be on the same scale as the binomial variables by dividing by two standard deviations[102] which allows for direct comparisons of the regression coefficients. We assessed the role of nutrition, coinfection by gastrointestinal nematodes & immunity in mediating the degree to which animals gained or lost schistosomes (change in CAA concentration). The variables considered were season (dry vs. wet), treated with anthelmintic bolus (yes vs no), age (continuous), CAA concentration(log), condition (continuous), IL4 (continuous), IFNy (continuous), hematocrit, total white blood cell count, eosinophil count, fecal egg count (FEC) of gastrointestinal nematodes (positive vs. negative), pregnancy status (yes vs. no), total globulins (continuous), days sample was in storage (continuous), total protein (continuous), plasma BKA, haptoglobin and fecal nitrogen (continuous). Backwards selection was performed dropping any term that did not improve AIC greater than 2 points. When we could no longer improve AIC by greater than 2 points we considered all models within that variation and the most parsimonious model (fewest terms) was selected as the final model.

#### Statistical analysis—Software

All generalized linear mixed models were run in R version 3.3.1, using the LME4and LMERtest packages[103,104] while model selection was done manually with the MASS package.

## Results

### Spatial and temporal variation in schistosome abundance

**Fig 1:**
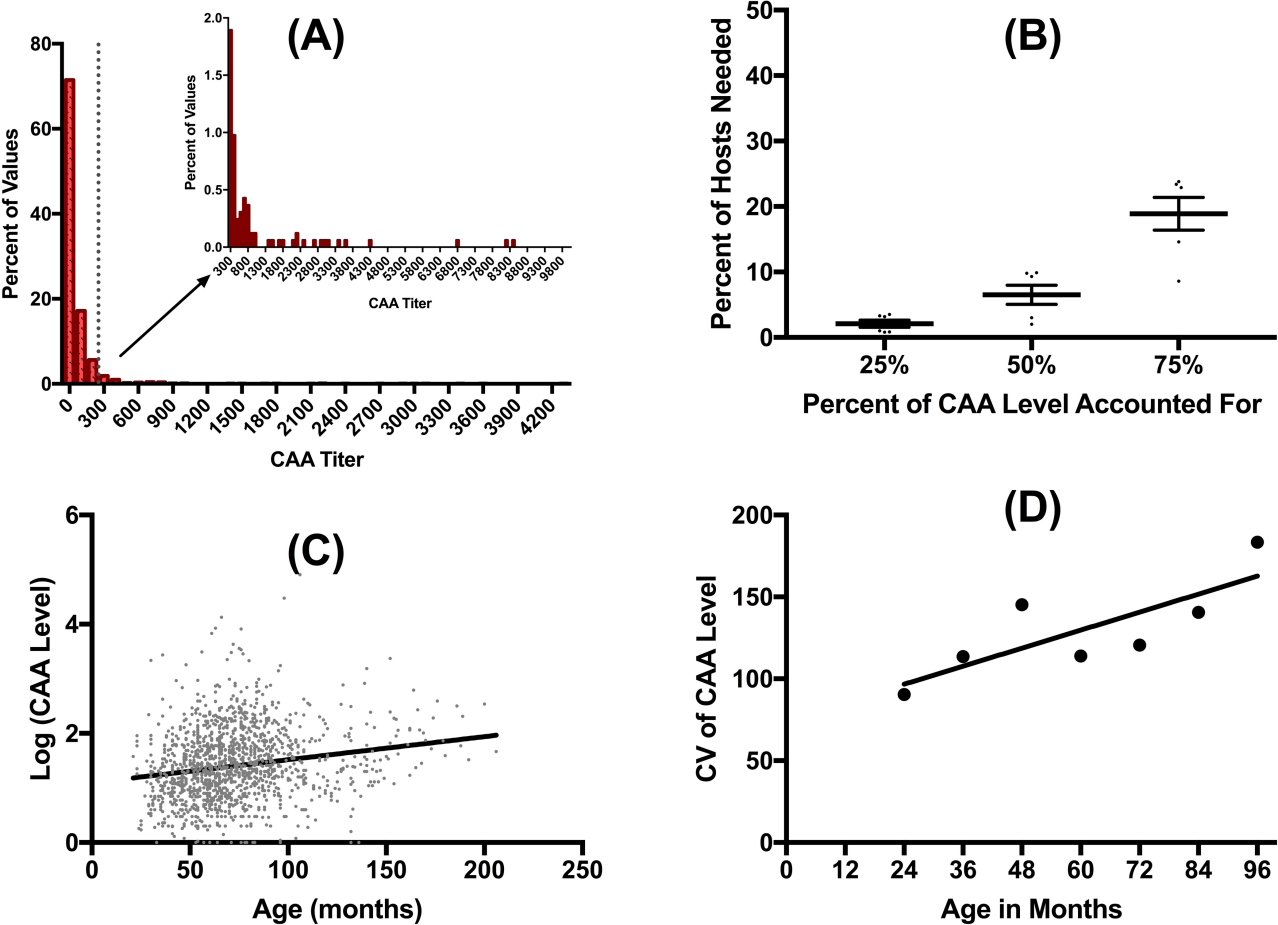
Schistosomes were highly aggregated (as measured by CAA level in pg/ml) with the majority of animals exhibiting very low levels (A). In fact, only 6.5% of hosts harbored approximately 50% of the worms (B). The percent of hosts needed to account for 25, 50 and 75% of the CAA level was calculated for each season and year (eg. wet 2009, dry 2009, wet 2010, dry 2010, etc) and each data point graphed here as mean +/- standard error (SEM). Schistosome burden increased with age (C) and the variability also increased (D). For variability, the coefficient of variation was calculated on CAA level for all individuals within a given year of age.

**Fig 2:**
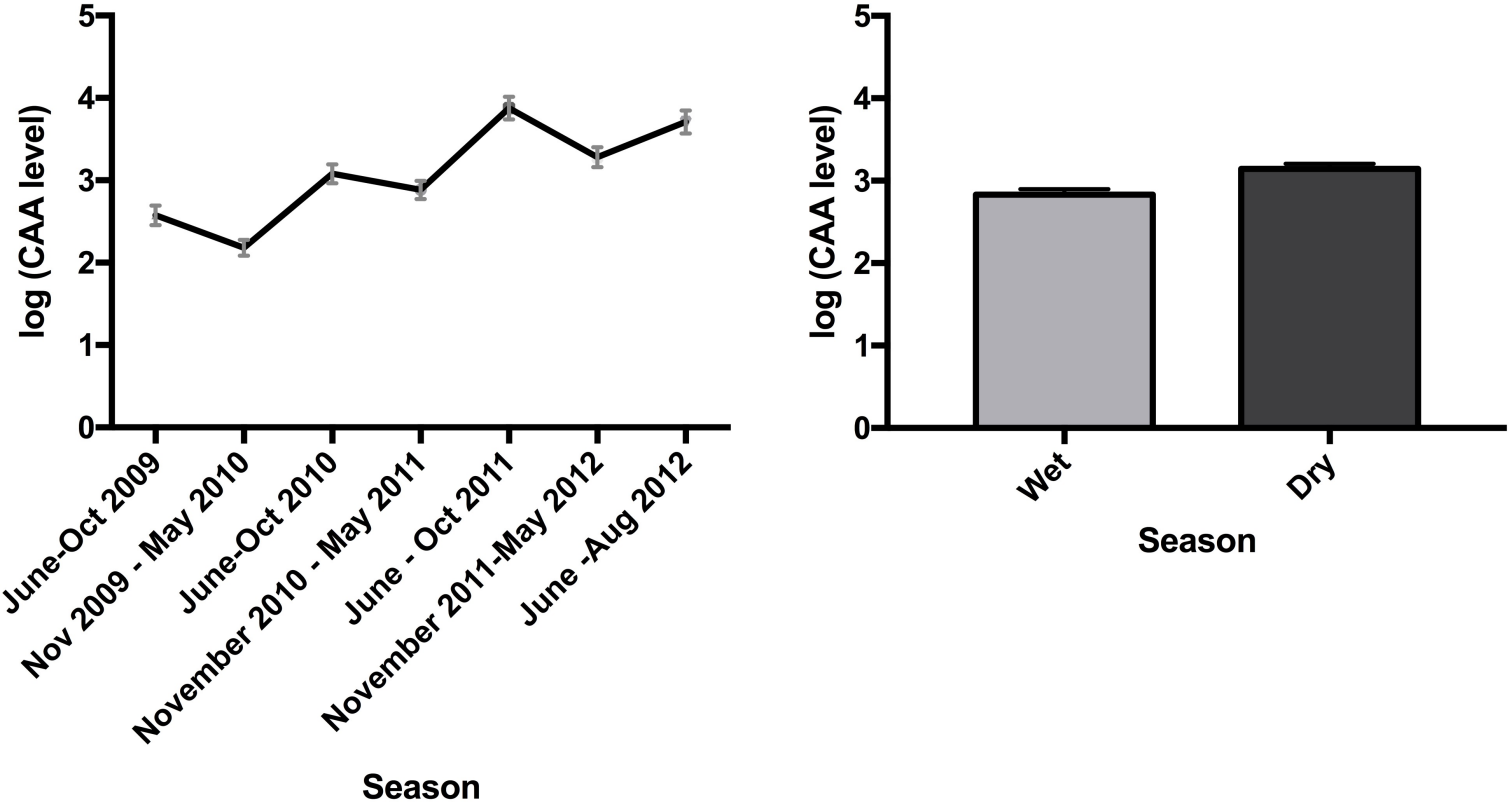
CAA level (pg/ml) increased over time (Panel A), with levels higher in the dry season (June to October) and lower in the wet season (November to May) (Panel B). Graphs show mean +/- SEM.

Schistosome infections in African buffalo were common, with an overall prevalence of 50%, and 79% of individuals exhibiting at least one positive test result (CAA>20) over the course of the study. Schistosomes were highly aggregated among hosts (Fig 1A), with most buffalo having very low concentrations at most time points (71% of the time animals had CAA concentrations below 100), and a small number of hosts harboring the majority of worms (Fig 1B). Schistosome burdens, and variability in burdens, increased with host age (Fig 1C and 1D). The intensity of schistosome infections varied seasonally, and among years (Fig 2, Table 2). Buffalo had higher schistosome burdens in the dry season than in the wet season. Overall, concentrations increased over the course of the study, with gains in schistosome numbers in the dry season only partially offset by losses during the following wet season. This increase likely reflects schistosome burdens in our study animals ratcheting up as they aged throughout the study: Replacing year with age in our analysis of spatio-temporal variation in schistosome burdens (Table 3) improves model fit (ANOVA p<0.05).

**Table 2:** Animals had higher schistosome levels in the dry season (June-October) than the wet season (November-May) and at Crocodile Bridge than at Lower Sabie after accounting for Animal ID as a random factor. Table shows the estimate from the GLMM with corresponding standard error (SEM) and p value.

*Model*: *ln(CAA)~Season+Initial Herd+Year(2008 reference)+(1|Animal ID)*	Estimate	SEM	p value	Associated Figure
Season (wet)	-0.23	0.13	0.07	Fig 3A
Herd (Lower Sabie)	-0.49	0.16	0.003	Fig 2B
Year 2009 (2008)	-0.45	0.20	0.03	Fig 3B
Year 2010 (2008)	-0.34	0.21	0.1	Fig 3B
Year 2011 (2008)	0.41	0.19	0.03	Fig 3B
Year 2012 (2008)	0.03	0.26	0.89	Fig 3B

**Table 3:** Older animals have higher burdens after accounting for Animal ID as a random factor, (model from 1A with age replacing year), however this pattern is driven by burdens in the dry season. Table shows the estimate from the GLMM with corresponding standard error (SEM) and p value.

*Model*: *ln(CAA)~Season+Initial Herd+age+(1|Animal ID)*	Estimate	SEM	p value	Interpretation
Age (rescaled)	0.98	0.15	<0.0001	Older animals have higher CAA levels, but this is driven by the dry season levels.
Age x Season	-0.45	0.18	0.01	
Season (wet)	-0.58	0.27	0.03	Buffalo have lower levels in the wet season.
Herd (Lower Sabie)	-0.66	0.18	0.0004	Buffalo at Lower Sabie have lower levels.

**Fig 3:**
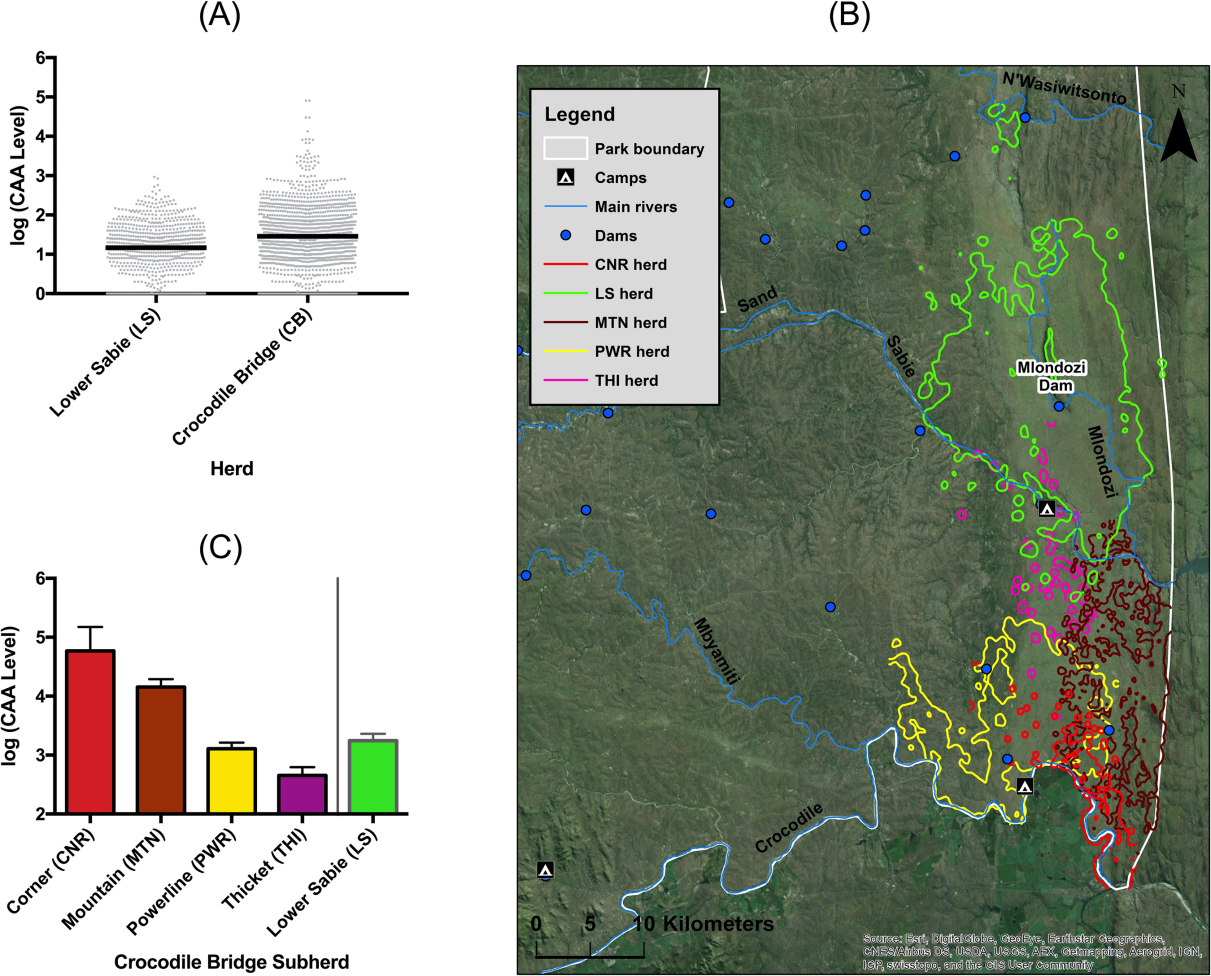
Buffalo at Crocodile Bridge have increased schistosome burdens compared to those at Lower Sabie (A), driven by animals in the two sub-herds located in the southeastern portion of the park (B&C). Graphs show mean CAA level (pg/ml) with SEM. The map in panel B shows the cumulative 95% utilization distribution of the buffalo herds under study.

There was pronounced spatial variation in schistosome abundances in our study population. Animals had higher intensities of infection at Crocodile Bridge than at Lower Sabie (Fig 3A, Table 2), which was due to two subgroups of the Crocodile Bridge herd in the southeastern corner of the park with high burdens (Fig 3B & Table 4).

**Table 4:** Animals in the corner and mountain subherds had higher schistosome burdens compared to the Lower Sabie herd, whereas there was no difference between the Powerline & Thicket subherds as compared to the Lower Sabie herd. Table shows the estimate from the GLMM with corresponding standard error (SEM) and p value.

*Model*:*lnSchisto~season+microherd+(1|Animal ID)+(1|Year)*	Estimate	SEM	p value
Season (wet)	-28.372	8.493	0.0001
Herd (corner)	114.801	29.21	0.0001
Herd (mountain)	69.05	19.84	0.006
Herd (powerline)	18.64	18.41	0.32
Herd (thicket)	-32.4	36.62	0.38

### Within-host schistosome dynamics

**Fig 4:**
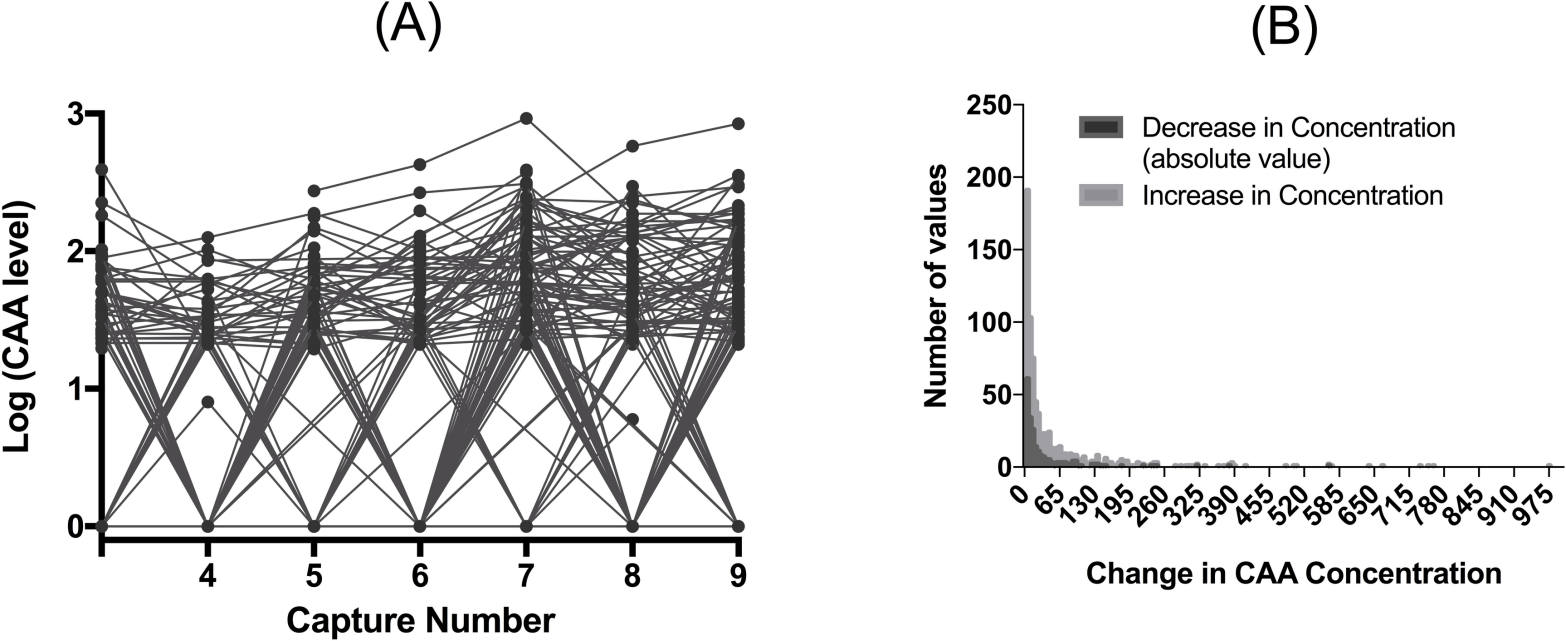
CAA levels (pg/ml) within an individual changed over time. In panel A we show the data for individuals with at least 4 consecutive capture points starting with the animals third capture (capture 2 has many missing data points). Any individual that is negative for CAA antigen (<20) we show the results as 0, consequently the line on the bottom represents multiple individuals. In panel B we show that the histogram of change in CAA level for timepoints where animals gained (increase in level) or lost worms (decrease in level).

Schistosome burdens varied dramatically over time within individuals (Fig 4). Animals both gained and lost schistosomes. The median decrease in CAA level was 12 (95% CI 9–15), while the median increase in CAA level was 15.7 (95% 13.1–18.5) however the range was large (Fig 4B). An increase in CAA concentration of 20 represents approximately 10 schistosome worms (S1 Text) with 11 animals having a decrease in of more than 50 worms in one 6-month period (Fig 4B). Animals were more likely to gain schistosomes in the dry season, and lose them in the wet season (model 4, binomial dependent variable = gain or loss of schistosomes; B = 0.57, SEM = 0.17, p = 0.009); and poor body condition was weakly associated with a higher likelihood of losing, rather than gaining, schistosomes (B = 0.31, SEM = 0.16, p = 0.06). No other terms were included in the final model.

**Fig 5:**
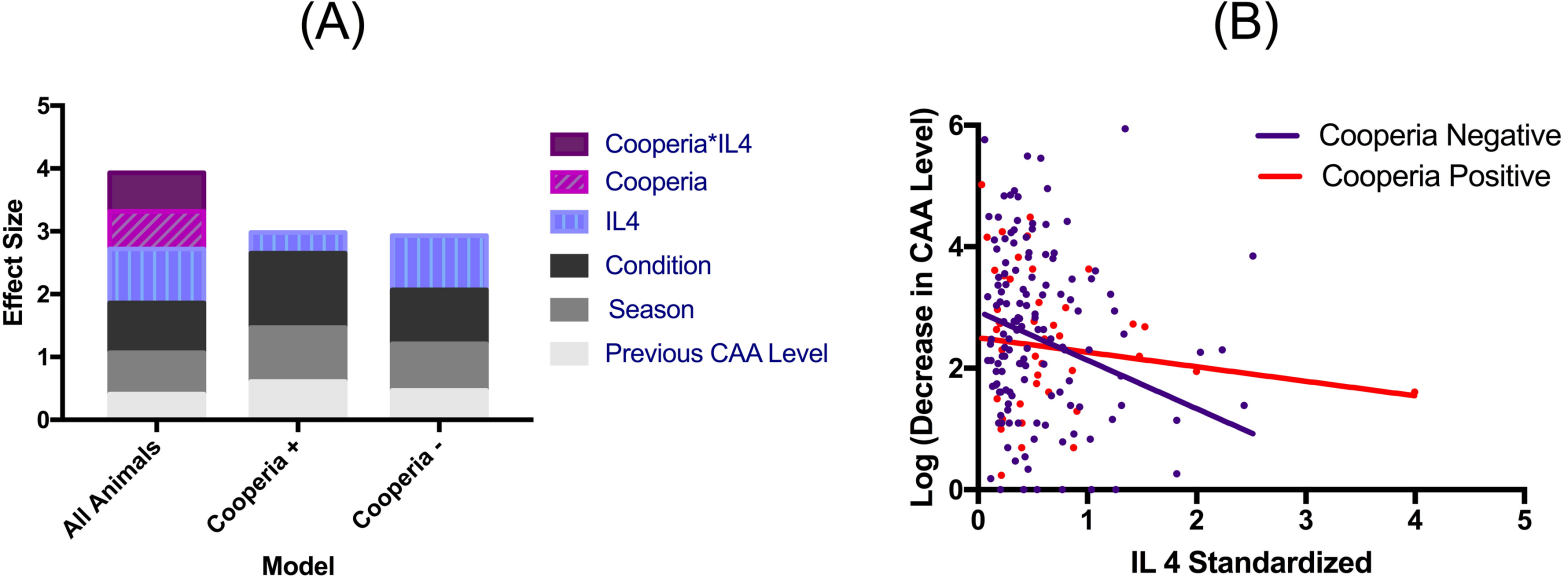
The largest predictor of whether animals lost schistosomes (as measured by CAA level in pg/ml) was the lack of an IL4 immune response, combined with coinfection (*Cooperia*) - but, host health (condition) and environment (season) also played a role (Panel A, overall with strongyle). Separating individuals based on *Cooperia* infection status reveals that IL4 concentration has a stronger (Panel A), negative relationship with schistosome burden in *Cooperia* negative individuals (Panel B).

We then considered what factors affected the magnitude of schistosome gain and loss (change in concentration) in individual hosts. The magnitude of schistosome gain was predicted only by season and the animal’s previous schistosome burden (model 5, Table 5). Buffalo gained more schistosomes in the dry season, and animals with prior high burdens tended to gain more additional worms. In contrast, greater losses of schistosomes were observed in the wet season, and occurred in buffalo in poor body condition, with lower levels of IL4, and those without gastrointestinal nematodes (model 6, Table 5). The effect of nematodes was driven by one species, *C*. *fuelleborni* (model 7, Table 6), with animals more likely to retain schistosomes if infected with this species. Additionally, the effect of IL4 on schistosome loss was strongest in animals that did not have *C*. *fuelleborni* while body condition had a much stronger effect on schistosome loss in those with this species of nematode (Fig 5). Pregnancy status, treatment with an anthelminthic bolus, fecal nitrogen, hematocrit, total white blood cell count, eosinophil count, total globulins, haptoglobin, plasma bactericidal ability, total plasma protein and age did not predict the size of an increase or decrease in schistosome number.

**Table 5:** After accounting for animal ID and subherd as random factors, the variables that affected the degree to which an animal loses schistosomes (model 6) were mostly mediated by factors important to disease progression such as immunity and health. However, the degree to which an animal gains (model 5) worms was predicted by environmental & host factors mediating exposure. Any variable with an NA means that variable was selected out of that model during model selection. Statistically significant variables (p<0.05) are denoted with a *.

	Schistosome number Decrease (n = 250)			Schistosome number Increase (N = 484)		
	Est	p value	Interpretation	Est	p Value	Interpretation
Log of Previous CAA-level	0.41	<0.001*	Animals lose more worms if they start with a higher CAA level.	0.43	<0.001*	Animals gain more worms if they start with a higher CAA level.
Age	-0.18	0.31	NS	0.18	0.15	NS
Season (wet)	-0.67	<0.001*	Worms are lost in the wet season	0.23	0.05*	Worms are gained in the dry season
Condition	-0.77	<0.001*	Animals in worse condition lose more worms.	NA	NA	NA
IL4	-0.88	<0.001*	Animals with less IL4 have a greater reduction in CAA level.	NA	NA	NA
Strongyle FEC (Positive)	-0.50	0.06	Animals without GI nematodes have a greater reduction in CAA level.	NA	NA	NA
IL4 x Strongyle FEC (Positive)	0.63	0.06	Animals with less IL4 have a greater reduction in CAA level only if the individual does not have GI nematodes.	NA	NA	NA

**Table 6:** The effect of gastrointestinal nematodes on the decrease in schistosome burden is driven by the presence of *C*. *fuelleborni*. *Haemonchus* presence is not retained in the model (model 7). Statistically significant variables (p<0.05) are denoted with a *.

Variable	Estimate	p Value
Log of Previous CAA Level	0.45	<0.001*
Age	-0.006	0.99
Season (reference = wet)	-0.87	<0.001*
Condition	-0.89	<0.001*
IL4	-0.85	<0.001*
*Cooperia* Presence (positive)	-1.23	0.002*
*Cooperia* Presence (positive) x IL4	0.78	0.06

## Discussion

### Spatial and temporal variation in schistosome abundance

Schistosome burdens varied seasonally and spatially in our study population of wild African buffalo. Conditions for schistosome exposure change radically between the wet and dry seasons. Buffalo, snail vectors, and larval schistosomes are water dependent and become concentrated at permanent water sources, such as slow moving rivers as the veld dries up[60,105]. During the wet season, buffalo spend less time watering in these permanent slow moving rivers, and can rely on other temporary water sources such as local pans where snail vectors are presumably less abundant[106]. Also, as water velocity in rivers increases, infection risk should decrease as snails and schistosome larvae are washed out. Indeed, infection dynamics of *S*. *mattheei* in snails have been found to be temporally variable and highly dependent on local conditions of both water flow and temperature which influence snail abundance and parasite development[58,107,108]. Seasonal change in schistosome prevalence in buffalo has been previously reported[109], with prevalence highest in the late dry season (58%) compared to the subsequent wet season (45%), a pattern consistent with our findings.

In addition to seasonal patterns of infection, we found variation in schistosome burdens among the herds studied. Buffalo live in fission-fusion societies with defined geographic ranges and frequent exchange of individuals between herds[69]. The populations we describe have high inter-group dispersal rates[77], and population genetic analyses suggest that there is little genetic sub-structuring among herds[110,111]. As such, it is unlikely that herd-level variation in schistosome burdens is based on genetic differences among herds. Instead, the observed spatial variation in worm burdens may be due to environmental heterogeneity that mediates exposure risk. We found the highest burdens in the herds that water along the eastern portion of the Crocodile River (mountain and corner herds), compared to those that water upriver, along the western portion of the river (powerline) or along the Sabie River/Mlondozi dam (thicket, Lower Sabie)[77]. The eastern part of the river is characterized by numerous manmade weirs that slow river flow and alter river profile (Govender, personal comm.) creating superior habitat for the snail intermediate host[106]. Concordant with these findings, Pitchford et al.[109] found that the highest egg output from buffalo occurred in streams or rivers with man-made dams and dense riparian vegetation. Thus, altered water flow due to dams and weirs may have helped establish schistosome transmission foci due to increased populations of vector hosts and retention of schistosome infective stages (e.g.[112]). In addition, there may be geographic variation in susceptibility to schistosomes, due to nutritional variability or localized differences in co-infecting parasite exposures. For instance, there is geographical variation in brucellosis[66] as well as some strongyle infections[93]; and the nutritional intake varies by locale with generally poorer body condition in the Crocodile Bridge herds[66].

### Within-host schistosome dynamics

Schistosome infections were highly dynamic in African buffalo. This longitudinal study showed that worm burdens in individual hosts fluctuated dramatically, but tended to increase over time, suggesting a lack of acquired immunity that is seen in other schistosome-host systems[29,54]. Schistosome burdens primarily increased during the dry season and decreased during the wet season, although typically not to baseline levels. The magnitude of schistosome clearance, with 11 animals losing more than approximately 50 worms, on this 6-month time scale, was not expected. In humans, schistosomes cause long-term, chronic infections lasting an average of 3–10 years[55,57,113,114] and even decades, due to molecular mimicry to prevent immune detection[29] and stem cells that repair damaged tissues—a trait schistosomes share with their planarian relatives that have incredible regeneration abilities[115]. Loss of adult worms or “self-cure” has been reported from Asian water buffalo, but is not well-known in humans[54,116,117]. The schistosome loss in African buffalo suggests a role for immune-mediated clearance of established schistosomes. It may be that in humans, loss of schistosomes also occurs on this short time scale, but is masked by continued recruitment of new parasites. Indeed, seasonal variation of schistosome burdens in humans has been reported from more temperate regions of their range where exposure is seasonal, suggesting significant worm loss in a 6-month period (e.g.[58,59,118]). However, the factors that cause natural worm loss in humans are unexplored even though death of worms within a host is thought to drive the development of acquired immunity because antigens that are normally hidden in a living worm become exposed upon death[23,119]. Our results are among the first to determine the ecological and host related factors that are associated with schistosome loss in a longitudinal study design.

Schistosome recruitment was primarily affected by variation in exposure but not variation in immunity. Of the host and environmental variables examined in this study, only season affected recruitment. Seasonal changes in vector and parasite infective stage abundance, and change in access to different water sources for the buffalo, likely underlie this pattern. Indeed, we have had difficulty finding snail vectors in the park during extreme dry conditions (Beechler pers obs.). Schistosome recruitment was also predicted by previous burden. Buffalo that previously had high schistosome burdens were likely to acquire more in the subsequent dry season, suggesting that certain individuals are more likely to gain worms than others. However, no host immune factors were associated with schistosome acquisition. Several species of hosts are known to develop partial, antibody-mediated immunity after repeated exposures to schistosomes, (e.g. humans, mice and cattle) although its strength varies among host species[23,24,120]. In humans, this response is IgE-mediated, is correlated with high levels of Th2 cytokines, like IL4, and removes migrating larvae via an antibody-dependent eosinophil response[29,121–123]. There was no evidence in our data that acquired immunity was providing protection against recruitment, as (i) IL4 and total globulins were not a significant driver of establishment and (ii) there was no age dependent effect such that older buffalo have lower prevalence or intensity of infection. On the contrary, worm burdens increased with host age. Taken together, these findings suggest that schistosome recruitment proceeds largely unhampered by acquired immunity. Nonetheless, it is possible that immune killing is localized in tissues such as the skin or lungs and thus systemic levels of immune factors are not reflective of local response. It is also possible that we “missed” acquired immunity in our study if it occurs in animals that are younger or older than those included in this analysis where the median age of buffalo at initial capture was 3 years.

By contrast, schistosome clearance was affected by immunological and physiological factors, and co-infection by gastrointestinal nematodes, as well as season. The strongest drivers of worm clearance were circulating levels of IL4 and host body condition. Buffalo with low levels of IL4, and buffalo in poor condition during the prior season, had fewer worms the following season. Although we predicted that a Th2 response would be associated with immune mediated clearing of worms, we found the opposite. Buffalo that had **low** IL4 levels during the previous collection period lost the most schistosomes by the next collection period. The pattern cannot be explained by schistosome immune polarization because, there was no association between simultaneous schistosome burden and IL4. If this were the case high burdens would be associated with IL4 and schistosome loss would be associated with a drop in IL4. Furthermore, The IL4 pattern does not appear to be due to a competing Th1 response as IFNy was eliminated during statistical model selection, and therefore not significant. It is possible that schistosome loss in buffalo is driven by Th2 immunity and due to the timing of our collection periods, we missed a peak in IL4 prior to schistosome loss, or that clearance occurs via an IL4-independent mechanism. Nevertheless, there remains a strong association between lower levels of IL4 during infection and worm loss. A seemingly parallel observation is that IL4 decreases to very low levels in water buffalo experimentally infected with its native schistosome, *Schistosoma japonicum[124]*. Although water buffalo are an important host for *S*. *japonicum*, they are not very permissive. Infections are short-lived, schistosome growth is reduced, and adaptive immunity develops over time[125,126]. However, in a more permissive host, yellow cattle, IL4 shows an increase after infection. Thus, comparing yellow cattle and water buffalo demonstrated that downregulation of IL4 is associated with worm expulsion and suggests that the immune response may help sustain infection[126]. Previous studies have indicated that an intact immune response, specifically CD4+ T cells are necessary for schistosome development and egg production in other host-schistosome models[127,128]. Therefore, we hypothesize that in buffalo, schistosomes require host signals that are associated with an Th2 response and are more likely to be lost when these host signals are not present.

Co-infecting gastrointestinal nematodes also influenced schistosome burden in that there was a significant association between schistosome loss and presence of gastrointestinal nematodes, which was primarily due to the presence or absence of one species of nematode, *C*. *fuelleborni*, and also an interaction between these nematodes and IL4. There was a negative association between schistosome loss and presence of *C*. *fuelleborni* such that buffalo with nematodes maintained more schistosomes and buffalo that did not have this nematode species lost more schistosomes. Several mechanisms may underlie this pattern. First, nematodes immunomodulate their hosts[129], including *Cooperia sp*.[54] and a previous study in cattle has shown that *Cooperia* sp. and a related gastrointestinal nematode, *Ostertagia ostertagi*, together drive an increase in host susceptibility to lungworms[130]. Thus, nematode-driven changes in buffalo immune response could be preventing immune mediated schistosome loss. Interestingly, our data indicate that the lack of IL4 is an important driver of schistosome loss in the absence of *C*. *fuelleborni*, however in the presence of this nematode species, IL4 is less important, suggesting that *C*. *fuelleborni* alters the immune-schistosome interaction. However, intensity of *C*. *fuelleborni* was not related to IL4 concentration in this population[99]. Second, buffalo that are naturally resistant to nematodes may also be good at reducing their schistosome burdens. Previous work in another African buffalo population has shown that there may be a genetic basis to variation in gastrointestinal nematode burdens, where some animals are naturally more effective at maintaining low nematode burdens than others[131]. This work also suggests that this phenotype is associated with variation in the immune response, specifically levels of IFNy secretion[131]. If similar genetically-based resistance patterns hold in KNP buffalo, then we hypothesize that the immune response that allows individuals to be resistant to nematodes also aids in clearance of schistosomes. Third, the *Cooperia*-schistosome interaction could be partially mediated by host condition. *Cooperia* is associated with increases in body condition[99], and, like the presence of *C*. *fuelleborni*, high body condition is associated with maintenance of schistosomes. However, buffalo condition was included (and significant) in the nematode-specific statistical analysis, suggesting that there is an *additional* effect of *C*. *fuelleborni* on schistosome infection.

Schistosomes were strongly influenced by the resource acquisition of their hosts. Resources directly affected parasite success and did not appear to increase immune clearance of schistosomes. Body condition of the buffalo was negatively associated with worm loss so that buffalo with low body condition scores (low resource acquisition) lost more worms, and high condition scores retained more worms. These data support the hypothesis that the schistosomes are highly dependent on host resources for success. Schistosomes feed on the blood of the host, ingesting red blood cells and directly absorbing nutrients like glucose across the tegument[42]. Although this may seem like a relatively unlimited resource, experimental studies have shown that malnutrition of the host retards egg development of schistosomes[38–41]; thus, they are nutritionally dependent on the host. During the dry season, high quality food is scarce and many buffalo become nutritionally deficient[61,66,91]. Thus, it is reasonable to hypothesize that missing components in the diet of buffalo during this period can lead to the starvation of worms, which are lost between the dry season and wet season when nutritious food is most scarce. We did not see evidence of resource acquisition enhancing host immunity, which is consistent with the observation that downregulated immune responses (i.e. decrease in IL4) was associated with worm loss. If our hypothesis that functioning Th2 immunity is necessary for schistosome maintenance is correct, then this adds a whole new angle to the theory regarding the effects of resource acquisition on pathogen dynamics, which as of yet has not considered the scenario where increased immune responses lead to parasite success (i.e. high resource acquisition positively affects the pathogen both directly and via the host immune system). It is possible that CAA levels are linked to changes in worm feeding as well as worm loss since it is an antigen produced by feeding worms; however, the magnitude seen here is typically associated with worm loss[132,133], not just reduced activity due to inadequate resources.

Our study demonstrates that schistosome infections are surprisingly dynamic in a free-living mammalian host population. Our longitudinal data on infection profiles of individual hosts over time uncover prominent heterogeneity in parasite dynamics with evidence for “self-cure” of schistosomiasis, the magnitude of which depends on host immunity, body condition and co-infection by gastrointestinal nematodes. On the other hand, we found no evidence for a role of host immunity mediating schistosome recruitment. Schistosome dynamics in free-living buffalo thus appear to be driven by successive waves of worm recruitment during the dry season, partly balanced by self-cure within a 6-month time frame. The consequences of these patterns for host health and disease dynamics are yet to be explored; but schistosome burdens in buffalo ratchet up with host age, and older individuals are thus likely to play a disproportionate role in schistosome transmission, as well as bearing the burden of schistosome-associated disease. Our results are in sharp contrast to previous work on schistosomiasis, where acquired immunity to schistosome establishment results in lower burdens in older animals, adult schistosomes are rarely killed by the host’s immune system, and effects of heterogeneity in host body condition and co-infections on parasite population dynamics are rarely considered. As such, studies in natural host populations may be indispensable for understanding which hosts are pivotal to parasite transmission and disease prevention, and focusing interventions accordingly.

## Supporting information

S1 Text CAA as a proxy for worm burden in African buffalo. (DOCX)

S2 Text Schistosome species identification. (DOCX)

S3 Text The effect of storage on immune measures. (DOCX)
