# Supplementary material for: Host immunity, nutrition and coinfection alter longitudinal infection patterns of schistosomes in a free ranging African buffalo population
Source: PLoS Negl Trop Dis. 2017 Dec 18;11(12):e0006122. doi: 10.1371/journal.pntd.0006122 (PMC5755937; doi:10.1371/journal.pntd.0006122)
Supplement: S1 Text — (DOCX) [file pntd.0006122.s001.docx]

**S1 Text: CAA as a proxy for worm burden in African Buffalo**

CAA is an antigen produced by metabolically active schistosomes. Previous studies using human patients also have shown that CAA levels significantly correlate with intensity of infection (Deelder *et al.* 1989a; De Jonge *et al.* 1988, Van Lieshout *et al.* 1992; Kremsner *et al.* 1993). However, for verification in buffalo, we correlated CAA levels with adult schistosome burdens determined upon necropsy.

During a cull, we visually examined a subsample of 15 buffalo (between July 2012-August 2012) for adult schistosomes in the intestinal mesentary. At cull, buffalo intestines were tied off at the duodenal-abomasal junction and the cecal-colic junction, and removed from the buffalo with all mesentery. They were then transported back to the laboratory and immediately evaluated for adult schistosomes (Figure A1a). Every schistosome was counted and collected into ethanol for species identification. We measured the CAA-concentration in plasma of all animals as described in the manuscript. For 15 animals, CAA-level and worm count were positively correlated (Spearman’s Rank coefficient = 0.52, p = 0.03, Figure A1b), showing that CAA concentration is a valid proxy for worm burden. We expect the manual counts to be less accurate than CAA titer, and while manual counts are a good approximation of intensity of infection it is unlikely that they represent entire burden. Manual counts of worms require that a person visually inspect the entire intestinal mesentery of a buffalo, which takes approximately 4-6 hours per buffalo during which time the schistosomes may migrate and move. As such we have performed a signed rank test, ranking animals from low to high burden by their count and correlating it to plasma CAA level. Numerically an increase in concentration of 20.66, represents an increase in 10 metabolically active schistosomes (SLR, Y = 2.296*X + 52.21).


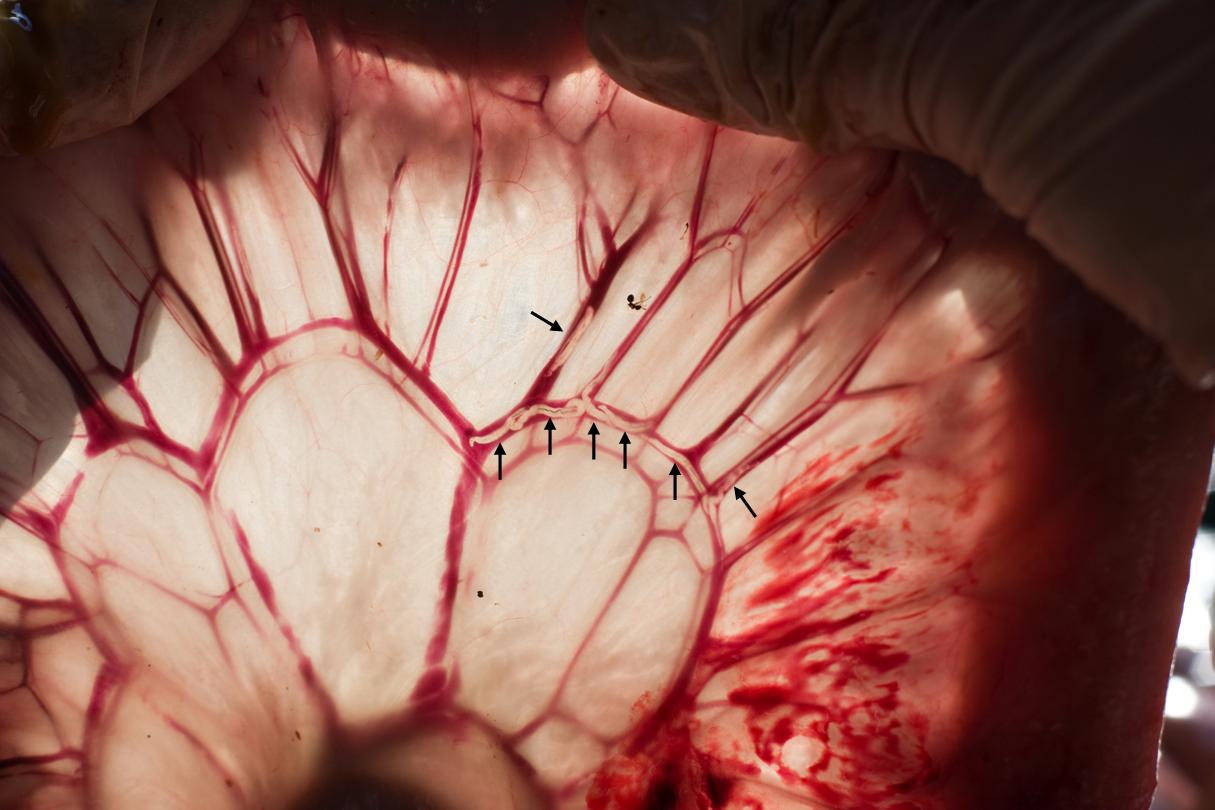


Figure S1: Schistosomes are shown in the mesenteric vessels of the intestines (a). The count of adult schistosomes correlated to CAA level (pg/ml) (b).

Literature Cited:

[Deelder, A.M., De Jonge, N., Fillié, Y.E., Kornelis, D., Helaha, D., Qian, Z.L., De Caluwé, P. & Polderman, A.M. (1989) Quantitative determination of circulating antigens in human schistosomiasis mansoni using an indirect hemagglutination assay. *The American journal of tropical medicine and hygiene*, **40**, 50–54.](http://paperpile.com/b/LFGRvD/ski8)

[De Jonge, N., Gryseels, B., Hilberath, G.W., Polderman, A.M. & Deelder, A.M. (1988) Detection of circulating anodic antigen by ELISA for seroepidemiology of schistosomiasis mansoni. *Transactions of the Royal Society of Tropical Medicine and Hygiene*, **82**, 591–594.](http://paperpile.com/b/LFGRvD/jm0b)

[Kremsner, P.G., de Jonge, N., Simarro, P.P., Mühlschlegel, F., Mir, M., Sima, F.O., Feldmeier, H., Bienzle, U. & Deelder, A.M. (1993) Quantitative determination of circulating anodic and cathodic antigens in serum and urine of individuals infected with Schistosoma intercalatum. *Transactions of the Royal Society of Tropical Medicine and Hygiene*, **87**, 167–169.](http://paperpile.com/b/LFGRvD/Dzgv)

[Van Lieshout, L., De Jonge, N., El Masry, N.A., Mansour, M.M., Krijger, F.W. & Deelder, A.M. (1992) Improved diagnostic performance of the circulating antigen assay in human schistosomiasis by parallel testing for circulating anodic and cathodic antigens in serum and urine. *The American journal of tropical medicine and hygiene*, **47**, 463–463.](http://paperpile.com/b/LFGRvD/cn3T)
