# Supplementary material for: Host immunity, nutrition and coinfection alter longitudinal infection patterns of schistosomes in a free ranging African buffalo population
Source: PLoS Negl Trop Dis. 2017 Dec 18;11(12):e0006122. doi: 10.1371/journal.pntd.0006122 (PMC5755937; doi:10.1371/journal.pntd.0006122)
Supplement: S2 Text — (DOCX) [file pntd.0006122.s002.docx]

**S2 Text: Schistosome species identification**

To confirm the identity of the schistosome collected from buffalo, we sequenced part of the large subunit ribosomal DNA (LSU) (4 individuals) and part of the 16S-12S region of the mitochondrial DNA (8 individuals). Single worms were lysed in a 5% chelex solution at ~99°C for 30 min. The lysate was used directly in a 10 µL PCR reaction following manufacturer’s instructions (GoTaq®, Promega, WI, USA) with the following primers: U178 (GCACCCGCTGAAYTTAAG) and L1642 (CCAGCGCCATCCATTTTCA) for the LSU (Lockyer et al 2003) and 16SF2 (GTGCTAAGGTAGCATAATAT) and 12SR2 (AACCGCGACTGCTGGCACTG) for 12S-16S (Morgan et al 2003). The thermocycling protocol was as follows: initial denature at 94°C for 3 min; 35 cycles of 94°C for 1 min, 50°C annealing for 30 sec, and 72°C for 1 min; and a final extension step at 72°C for 7 min. The samples were visualized along with negative controls on a 1% agarose gel and successful samples were cleaned up with the E.Z.N.A. Cycle Pure kit (Omega Bio-tek, GA, USA) and submitted to GenScript (NJ, USA) for sequencing. Nucleotide BLAST® searches were performed to find the most similar organisms in GenBank.

The LSU sequences were 820 bp and were 100% identical to each other and 100% to *Schistosoma mattheei* in GenBank (AY157265.1). This region is known to differ among species of schistosomes and is phylogenetically informative (Kane et al 2003). The mitochondrial DNA sequences were 100% identical to each other and 99% identical to *S. mattheei* (accession: [LM209780.1](http://www.ncbi.nlm.nih.gov/nucleotide/689716978?report=genbank&log$=nucltop&blast_rank=1&RID=98HCCN8E01R)) from genome assembly  [emb|LM209780.1](http://www.ncbi.nlm.nih.gov/nucleotide/689716978?report=genbank&log$=nuclalign&blast_rank=1&RID=98HCCN8E01R) with a score of 985 and 78% coverage. The next most similar sequences were that of *S. curassoni* and *S. haematobium* with 90% identity. These species are close relatives of *S. mattheei* (Lawton et al 2011).

Lockyer AE, Olson PD, Ostergaard P, Rollinson D, Johnston DA, Attwood SW, Southgate VR, Horak P, Snyder SD, Le TH *et al*: **The phylogeny of the Schistosomatidae based on three genes with emphasis on the interrelationships of *Schistosoma* Weinland, 1858**. *Parasitology* 2003, **126**:203-224.

Morgan JAT, DeJong RJ, Lwambo NJS, Mungai BN, Mkoji GM, Loker ES: **First report of a natural hybrid between *Schistosoma mansoni* and *S. rodhaini***. *J Parasitol* 2003, **89**(2):416-418.

Kane RA, Southgate VR, Rollinson D, Littlewood DTJ, Lockyer AE, Pages JR, Tchuem Tchuenté LA, Jourdane J: **A phylogeny based on three mitochondrial genes supports the division of *Schistosoma intercalatum* into two separate species**. *Parasitology* 2003, **127**:131-137.

Lawton SP, Hirai H, Ironside JE, Johnston DA, Rollinson D: **Genomes and geography: genomic insights into the evolution and phylogeography of the genus *Schistosoma***. *Parasite Vector* 2011, **4**.
