# Supplementary material for: Host immunity, nutrition and coinfection alter longitudinal infection patterns of schistosomes in a free ranging African buffalo population
Source: PLoS Negl Trop Dis. 2017 Dec 18;11(12):e0006122. doi: 10.1371/journal.pntd.0006122 (PMC5755937; doi:10.1371/journal.pntd.0006122)
Supplement: S3 Text — (DOCX) [file pntd.0006122.s003.docx]

**S3 Text: The effect of storage on immune measures**

**Plasma BKA:** To optimize the plasma BKA for African buffalo and for use in our laboratory we first ran a bacterial growth curve to demonstrate that the plateau of killing occurred between 9-10 hours after plating, and was stable until 24 hours. Following this a plasma dilution was selected by testing 2 fold dilutions from 1:2 to 1:128 with 20 randomly selected samples with a target area of 1:2-1:8 selected because approximately 50% of *E. coli* were killed. A second set of 20 randomly selected samples were run at dilutions of 1:2, 1:3, 1:5, 1:8 and the dilution of 1:3 was selected because it had the greatest repeatability (intraplate CV<10%) with mean killing activity of 52%. Since samples were run several years after collection (samples were evaluated November 2014) we evaluated whether storage time (months in freezer) correlated to killing ability, but no correlation was found using a simple linear regression (F=0.31, p=0.5740).

**Total globulins:** Total globulins (alpha, beta & gamma) were assessed using an Abaxis chemistry panel from sample stored at -20 ºC until the time of testing (June-August 2014) (Couch et al, in review). Since samples were run several years after collection we evaluated whether storage time (months in freezer) correlated to globulin concentration using a simple linear regression (F=26.13, p<0.001). Since it decreased with time we used time in storage as a covariate in all analyses with globulins.

**Haptoglobin:** Haptoglobin was evaluated from stored plasma samples (stored at -20 ºC) and all testing was performed in November 2014 using a commercially available ELISA kit for bovine haptoglobin (Life Diagnostics, product number HAPT 11). Since samples were run several years after collection we evaluated whether storage time (months in freezer) correlated to log(haptoglobin concentration) using a simple linear regression but no correlation was found (SLR, F=0.1467, p=0.708). Log transformation was used to normalize the distribution for analysis.
